# Physical health promotion for young people at ultra‐high risk for psychosis: An application of the COM‐B model and behaviour‐change wheel

**DOI:** 10.1111/inm.12243

**Published:** 2016-07-19

**Authors:** Rebekah Carney, Tim Bradshaw, Alison R. Yung

**Affiliations:** ^1^ Institute of Brain, Behaviour and Mental Health University of Manchester Manchester UK; ^2^ School of Nursing, Midwifery and Social Work University of Manchester Manchester UK; ^3^ Greater Manchester West Mental Health NHS Foundation Trust Manchester UK

**Keywords:** lifestyle, mental health nursing, physical health, ultra‐high risk

## Abstract

People with psychotic illnesses, such as schizophrenia, have high rates of unhealthy lifestyle factors, such as smoking and physical inactivity. Young people who seek help for mental health care, particularly those at high risk for psychosis, often also display high rates of these unhealthy behaviours. Although healthy living interventions have been applied to people with established psychosis, no attempt has been made to offer them to young people at risk for developing psychosis, despite potential benefits to mental and physical health. We propose that the COM‐B model (consisting of capability, opportunity and motivation) and behaviour‐change wheel might be an appropriate framework that mental health nurses and other health professionals could apply. Using a systematic and theoretically‐based approach to intervention development could result in effective methods of health promotion in this group. Further training and development for mental health nurses could encourage a greater integration of mental and physical health care.

## Introduction

The physical health disparities of people with schizophrenia and severe mental illness are an area of increasing concern (Shiers *et al.* 2015). Together with the unwanted side‐effects of antipsychotic medication, this increased morbidity is partly due to adverse lifestyle factors, including tobacco use, poor diet, and physical inactivity (Addy *et al.* 2012; Hennekens *et al.* 2005; Vancampfort *et al.* 2013). However, these lifestyle factors are also present from an early stage (first‐episode of psychosis (FEP)), and might even occur prior to the onset of psychosis, in those who are at ultra‐high risk (UHR) for psychosis or putatively ‘prodromal’ (Addington *et al.* 2015; Carney *et al.* 2016).

UHR individuals (otherwise referred to as the ‘at‐risk mental state’ (ARMS) group are characterized by the presence of attenuated psychotic symptoms, brief limited intermittent psychotic symptoms that spontaneously resolve, or genetic‐risk combined with recent decline in functioning (Yung *et al.* 2004). Within 3 years, we can expect approximately one‐third of UHR individuals to progress to a full‐threshold psychotic illness, with a large proportion developing schizophrenia (Nelson *et al.* 2013). Other psychological illnesses are also prevalent in this group, such as anxiety, mood, or substance use disorders (Addington *et al.* 2011; Lin *et al.* 2015), and many continue to function poorly, regardless of symptomatic remission (Cotter *et al.* 2014).

Existing interventions for UHR cohorts focus primarily on addressing poor mental health and providing supportive psychological therapies to prevent the onset of psychotic illnesses (van der Gaag *et al.* 2013; Yung *et al.* 2011). Physical health and lifestyle behaviours are generally not addressed or monitored routinely in services (Carney *et al.* 2015), and to date, healthy lifestyle interventions have not been applied to this group. This is despite potential benefits to future physical and mental health. First, if a UHR individual develops a FEP, he or she will almost certainly receive antipsychotic medication. The side‐effects of antipsychotics might then further exacerbate already compromised physical health due to the unhealthy lifestyle factors noted earlier (De Hert *et al.* 2006). Second, even if a psychotic illness does not develop, encouraging a healthy lifestyle might protect against future ill health and health implications arising from these unhealthy behaviours, such as continued tobacco use. Finally, there is evidence to suggest that high rates of unhealthy lifestyle factors could contribute to the onset of psychosis. This includes the use of high‐potency cannabis (Di Forti *et al.* 2014), tobacco (Gurillo *et al.* 2015), and physical inactivity (Koivukangas *et al.* 2010). Therefore, the UHR group represents an important target for health professionals when applying healthy lifestyle interventions. Mental health nurses might be an appropriate target to deliver such interventions, as they are frequently in contact with service users and are seen as having a holistic role in managing mental and physical health (Bradshaw and Pedley, 2012).

### Unique Characteristics of the UHR Group

In addition to subthreshold psychotic symptoms, UHR individuals frequently report depression and anxiety (Fusar‐Poli *et al.* 2013; Yung *et al.* 2004). Although they are usually no different to their peers with regards to weight and body composition, they do have high levels of unhealthy lifestyle behaviours (Addington *et al.* 2015; Carney *et al.* 2016). Designing an intervention must take these characteristics into account. However, issues which usually have to be considered when developing lifestyle interventions for people with schizophrenia might not be relevant, such as long‐standing illness, high levels of negative symptoms, cognitive impairment, and antipsychotic side‐effects, such as weight gain and metabolic disturbance. We aimed to assess which behaviour‐change theories and techniques might be useful to underpin a healthy living intervention for the UHR group, given their unique characteristics. We also aimed to discuss how this can be related to clinical practice, and how mental health nurses might have an important role in promoting physical health.

### Existing Interventions

Happell *et al*. (2012) discuss how physical health interventions are required to promote the well‐being of people with mental health difficulties. A focus on developing health‐behaviour interventions within the nursing and wider health‐care professions might encourage significant benefits in the general health of young people who use mental health services. One approach recommended by the Medical Research Council (Craig *et al*. 2008) is to use a theoretical background to develop methods of health promotion. A sound theoretical framework will assist the delivery of an intervention through the application of a well‐developed structure, based on the best available evidence (Hillsdon *et al*. 2004). The alternative is to simply apply interventions in the hope that they might work. However, behaviour change is a complex area, with many overlapping concepts and theories. The most common social cognition models applied to behaviour‐change research are social learning theory (Bandura, 1986), the Health Belief Model (Rosenstock, 1974), theory of planned behaviour (Ajzen, 1991), and the Transtheoretical Model (Prochaska and Velicer, 1997).

These models are useful to predict and explain human behaviour; however, their application to behaviour‐change interventions is less clear. National Institute for Health and Care Excellence (NICE) guidelines (2007) state that evidence supporting any specific psychological model to inform behaviour change is limited (Abraham *et al.* 2009). A recent meta‐analysis concluded that the relationship between the use of theory and effectiveness of an intervention is weak (Prestwich *et al.* 2014). Additionally, despite MRC (Craig *et al*. 2008) recommendations, many health‐behaviour interventions lack a clear and specific theoretical foundation (Davies *et al.* 2010; Hardeman *et al.* 2002; Prestwich *et al.* 2014). Even those that include a theoretical model often fail to link the mechanisms of change back to theoretical constructs (Michie and Prestwich, 2010; Prestwich *et al.* 2014). This could be due to the inconsistency in existing frameworks when describing how to apply theory to an intervention.

### Promoting Behaviour Change

Creating an intervention involves the initial difficulty of identifying which behaviour should be targeted to achieve a desired health outcome. For example, in a healthy living intervention for young people, the aim might be to promote weight loss, for which many types of behaviours could be targeted, including dietary intake, physical activity, and sedentary behaviour. Once a behaviour is identified, techniques to promote this change can be applied.

Behaviour‐change techniques (BCT) are the active components which make up an intervention, and are observable and replicable (Michie and Johnston, 2012). The behaviour‐change technique taxonomy consists of 93 'active ingredients' that can be used to facilitate behaviour change (Abraham and Michie, 2008; Michie and Johnston, 2012); for example, the use of goal setting and the provision of instructions in a physical activity intervention. Individual taxonomies have also been refined containing a smaller set of techniques, which could be most effective for smoking cessation (Michie *et al.* 2011b), or interventions for physical activity and diet (Michie *et al.* 2011a), and alcohol consumption (Michie *et al.* 2012).

NICE suggests a number of BCT that might be effective for driving behaviour change, including self‐monitoring techniques (NICE, 2007; NICE, 2014). Self‐monitoring involves recording behaviours (i.e. keeping a food diary), setting goals, and obtaining feedback (Abraham and Michie, 2008). NICE (2014) also suggest applying these techniques using remote methods via text messaging or mobile apps (NICE, 2014). Self‐monitoring techniques are recommended for the general population (Michie *et al.* 2009), obese adults and those with obesity related comorbidities (Dombrowski *et al.* 2012), and people with recently‐diagnosed diabetes (Hankonen *et al.* 2014).

### Interventions For Clinical Populations

NICE guidelines (2014) emphasize that techniques used in lifestyle interventions should match service users' needs. Therefore, the characteristics of the UHR cohort need to be considered to ensure any new lifestyle interventions are appropriate. As noted earlier, health promotion for UHR individuals is currently limited. While interventions designed for the general population might not be appropriate, neither might those developed for people with schizophrenia. Given that UHR individuals frequently experience high levels of depression (Fusar‐Poli *et al.* 2014; Yung *et al.* 2004), we examined the techniques commonly used in populations with clinical depression to promote physical activity.

A recent Cochrane review examined the effectiveness of exercise interventions for depression (Cooney *et al.* 2014). Individuals with clinical depression from inpatient or community outpatient settings usually receive supervised, guided exercise sessions conducted by a professionally‐qualified physical activity trainer (Carta *et al.* 2008; Knubben *et al.* 2007; Martinsen *et al.* 1985; Mota‐Pereira *et al.* 2011; Pilu *et al.* 2007; Schuch *et al.* 2011). Individuals are also given information about correct exercise technique (Knubben *et al.* 2007), and receive positive feedback from trainers throughout sessions (Carta *et al.* 2008; Pilu *et al.* 2007). However, it is unclear whether other BCT are employed, as many studies lack sufficient detail for further analysis.

As UHR individuals present with subthreshold, emerging psychotic symptoms, we also considered physical activity interventions for people with early psychosis. Although there are many such interventions for people with schizophrenia (Firth *et al.* 2015), to date, only one review has assessed the active components of physical activity interventions for first‐episode samples (Rosenbaum *et al.* 2014). Similar to interventions for people with depression, the most common techniques employed are the provision of information and guidance about exercise (Alvarez‐Jimenez *et al.* 2006; Abdel‐Baki *et al.* 2013; Curtis *et al.* 2015a; Fredrikson *et al.* 2014; Killackey *et al.* 2011; Lin *et al.* 2011; Lovell *et al.* 2014; Smith *et al.* 2014), supervised sessions (Curtis *et al.* 2015a; Lin *et al.* 2011; Smith *et al.* 2014), and the use of goal setting (Fredrikson *et al.* 2014; Killackey *et al.* 2011). A further qualitative study found FEP individuals value the peer support of a training partner and an individualized approach to designing an exercise intervention (Firth *et al.* 2016).

Common techniques used for FEP populations and individuals with depression are the provision of a professionally‐qualified trainer who is available to supervise, demonstrate, and give instructions during exercise sessions. Therefore, increasing the opportunity to exercise by providing facilities, or allocating time for physical activity and enhancing a person's knowledge so they feel capable to exercise, might be particularly useful. Nonetheless, we should look further than the methods used for these patient groups, as they might not be the only effective techniques to employ for UHR individuals. Additionally, many existing interventions are vague and do not provide sufficient detail for replication.

### COM‐B Model of Behaviour and Behaviour‐Change Wheel

Despite MRC (Craig *et al*. 2008) recommendations of using theory to guide intervention development, little advice is given on how mental health nurses and allied health professionals can apply theory to behaviour‐change interventions in practice, and how health professionals can select the most appropriate techniques to use. The behaviour‐change wheel is a new framework that aims to promote a systematic method of intervention development, (Michie *et al.* 2011c; Michie *et al.* 2015).

**Figure 1:**
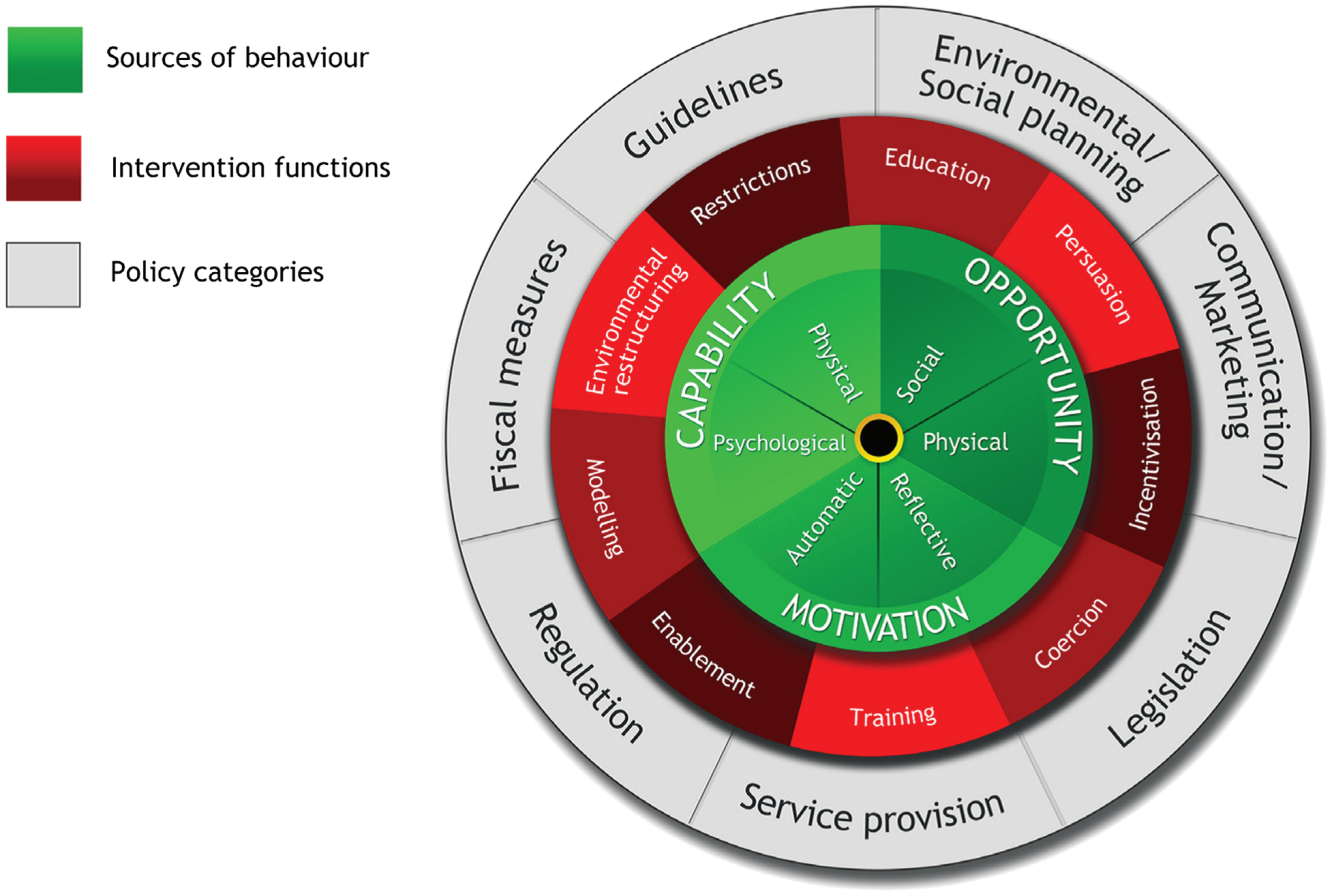
Behaviour change wheel Michie et al. (2011c). [Colour figure can be viewed at http://wileyonlinelibrary.com]

The behaviour‐change wheel has been described elsewhere (Michie *et al.* 2011c; Michie *et al.* 2015). To summarize, the behaviour‐change wheel is made up of three layers (Fig. 1). At the centre of the framework sits a theoretical model that proposes three ways in which a behaviour occurs: capability, opportunity, and motivation (the COM‐B model; Michie *et al.* 2011c; Michie *et al.* 2015). This helps identify which source of behaviour should be targeted. Surrounding the COM‐B model are intervention categories that provide methods to promote behaviour change and include education, persuasion, incentivization, coercion, training, restriction, environmental restructuring, modelling, and enablement (see Table 1 for examples and definitions from Michie *et al*. (2011c)). The final layer of the behaviour‐change wheel contains policy categories showing how intervention functions cab be applied on a wider scale (Michie *et al.* 2015).

**Table 1:** Possible intervention functions to encourage a healthy lifestyle in UHR cohorts

Intervention class	Definition (from Michie *et al.* 2011, p7)	Example applied to a healthy living intervention for the UHR cohort
Education	Increasing knowledge or understanding	Provide instruction about exercise, increase understanding of why it is important for physical health.
Persuasion	Using communication to induce positive or negative feelings or simulate action	Discussing the benefits of a healthy lifestyle, such as improved mood, more energy; and provide general encouragement when behaviour is carried out.
Incentivization	Creating expectation of reward	Encourage goal setting, such as 2 hours per week exercise and allocating rewards when goals are met.
Coercion	Creating expectation of punishment or cost	Provide information about consequences of unhealthy habits; for example, smoking increases cancer risk.
Training	Imparting skills	Encourage the relevant skills to be developed that enable a person to be able to exercise, such as gym training.
Restriction	Using rules to reduce the opportunity to engage in the target behaviour (or to increase the target behaviour by reducing the opportunity to engage in competing behaviours)	Increase the minimum price of alcohol frequently used to target younger populations, such as alcopops.
Environmental restructuring	Changing the physical or social context	Private gym, diet, or behavioural support classes for young people who are taking part in the intervention with a trainer who has awareness of mental health.
Modelling	Providing an example for people to aspire to or imitate	Using a gym buddy system where UHR individuals are accompanied to exercise sessions by a peer or staff member.
Enablement	Increasing means/reducing barriers to increase capability or opportunity	Prompt practice of exercise sessions or cooking sessions, accompany an individual to gym until they feel confident to go alone.
Policy		
communication/marketing	Using print/electronic/telephonic or broadcast media	Develop leaflets and materials to be used in the UHR service, which educate people about living a healthy lifestyle or provide warnings to stop smoking.
Guidelines	Creating documents that recommend or mandate practice, including changes to service provision	Ensure young people who access UHR services have a physical health assessment and are given information about their health.
Fiscal	Using the tax system to reduce or increase the financial cost	Increase taxation on tobacco and high‐sugar products.
Regulation	Establish rules or principles of behaviour or practice	Reduce adverts for fast food in areas populated by young people such as around colleges and replace with healthy food or gyms.
Legislation	Making or changing laws	Enforce limits on the amount of alcohol one person can buy if under the age of 21 years.
Environmental/social planning	Designing and/or controlling the physical or social environment	Encourage local areas to have accessible facilities, such as gyms and green spaces.
Service provision	Delivering a service	Encourage parity of esteem in mental health services.

UHR, ultra‐high risk for psychosis.

Although a relatively new model, the COM‐B model has been successfully applied as a framework to the English Department of Health 2010 tobacco control strategy (Health, 2010), the NICE guidance on reducing obesity (NICE, 2006), medication adherence and management (Jackson *et al.* 2014; Sinnott *et al.* 2015), management of spinal cord injury (Bérubé *et al.* 2015), childhood obesity (Curtis *et al.* 2015b; Robinson *et al.* 2013), and promotion of safe‐sex practices (Newby *et al.* 2013). It has also recently been adopted by Improving Access to Psychological Therapy teams to guide the application of low‐intensity lifestyle interventions for psychological well‐being practitioners and other health professionals.

### Application of the COM‐B model to a UHR cohort

**Figure 2:**
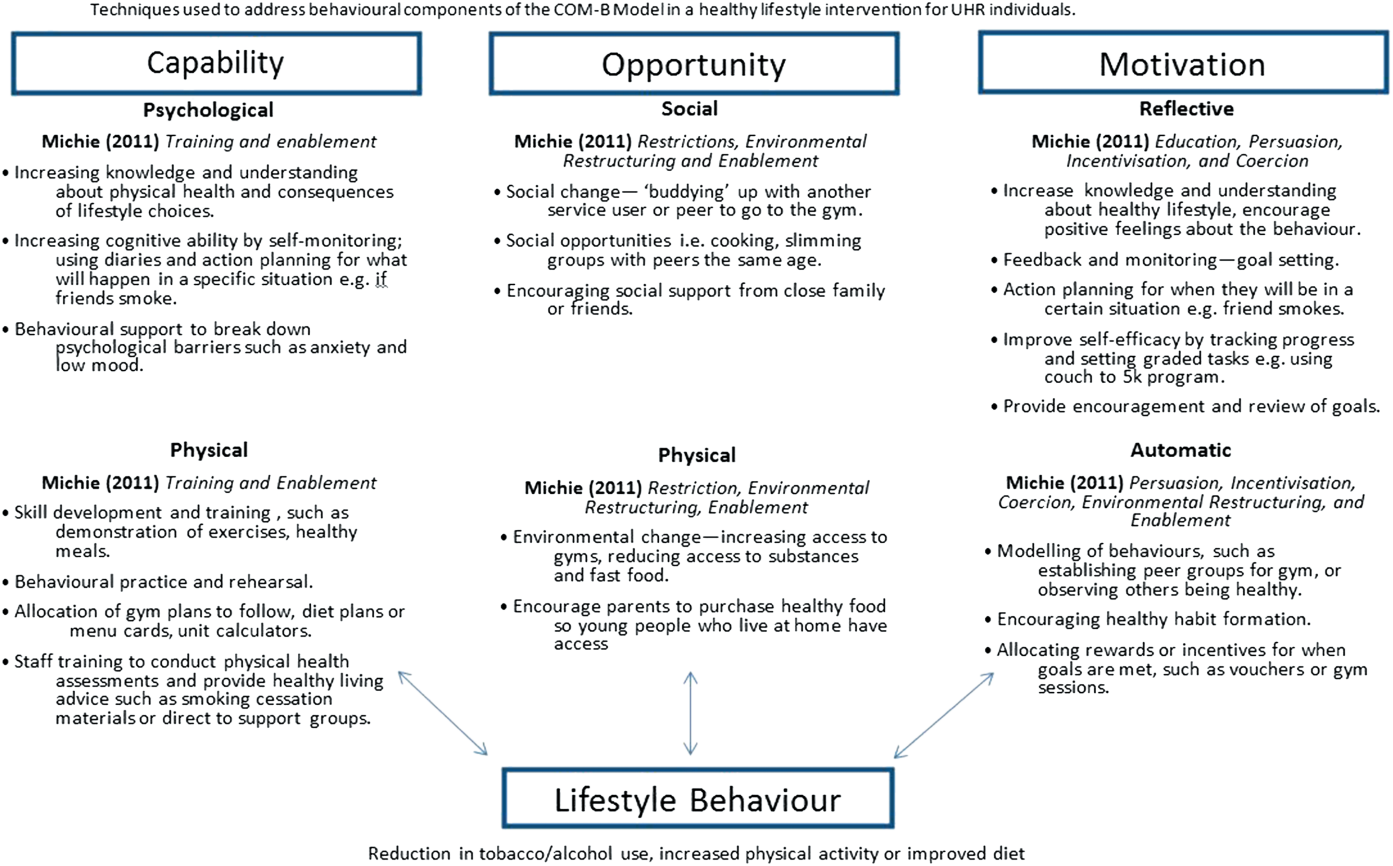
Techniques used to address capability, opportunity and motivation from the COM‐B model. [Colour figure can be viewed at http://wileyonlinelibrary.com]

We propose that the COM‐B model could be useful to develop a lifestyle intervention to promote the physical health of the UHR group. It provides a systematic and standardized approach to developing an intervention, and allows theoretically‐based BCT to be applied to guide behaviour change. Using clearly‐defined techniques proposed by the COM‐B model and behaviour‐change wheel taxonomy will ensure transparency and enable replication of an intervention (Abraham and Michie, 2008; Michie *et al.* 2011c). To date, there have been no published or recorded physical health interventions for the UHR group. We suggest that using a theoretically‐based framework to develop an intervention will provide a good baseline to conduct further research and develop health‐service provision for this population. In the present study, we outline how each of the three components of the behaviour‐change wheel could be addressed for the UHR cohort (Fig. 2).

### Motivation

Amotivation or avolition is observed in some young UHR individuals, and can impact on a person's daily functioning (Piskulic *et al.* 2012). Targeting motivation according to the COM‐B model could involve increasing knowledge about exercise and diet, and discussing the benefits of living a healthy lifestyle. Goal setting and self‐monitoring, such as aiming for two gym sessions per week or eating five servings of fruit and vegetables daily, recorded in a diary might increase reflective motivation.

The concept of self‐efficacy has an important influence on motivation (Bandura, 1977; Schunk, 1995; Zimmerman *et al.* 1992). Self‐efficacy refers to an individual's belief in their own capacity to engage in a given behaviour (Bandura, 1977; Bandura, 1982). Motivation is enhanced when people have a greater sense of competency and self‐belief that they can complete a task (Schunk, 1995; Zimmerman *et al.* 1992). Empirical evidence suggests self‐efficacy has an important role in determining whether an individual engages in a given health behaviour and their motivation to change that behaviour (Holloway and Watson, 2002; Thirlaway and Upton, 2009). Programmes targeting self‐efficacy to increase healthy eating in adolescents are effective in improving dietary choices and increasing physical activity in adults (Fitzgerald *et al.* 2013; Lee *et al.* 2008; Olander *et al.* 2013). Despite this, self‐efficacy has not been explored in UHR individuals. However, in people with schizophrenia, a lack of self‐belief and low physical competence (i.e. low self‐efficacy) is related to physical inactivity (Vancampfort *et al.* 2011). Therefore, self‐efficacy could be an appropriate target for the UHR group.

### Opportunity

Considering environmental factors and social opportunities could prove useful when developing interventions for the UHR group, as poor social environment, deprivation, and other socioeconomic factors are risk factors for psychosis, and UHR individuals tend to live in socially‐deprived areas (Allardyce *et al.* 2005; Cotter *et al.* 2015; O'Donoghue *et al.* 2015). Working with young people to change the context in which a behaviour usually does (or does not) occur might be helpful to promote a healthy lifestyle. For instance, if a person lives in a deprived area, rather than encouraging them to exercise outdoors, sessions could be conducted in local gyms in a safe environment. Thus, the environmental setting can be developed to be as conducive to an individual as possible. A supportive social environment could be created by conducting small group sessions with other participants who are also motivated to improve their physical health.

### Capability

Enhancing psychological capability might focus on breaking down some of the psychological barriers young people experience. This could include providing behavioural support for low mood and high levels of anxiety experienced by UHR individuals. Physical capability could also be targeted using education and training intervention functions to demonstrate correct exercise techniques, such as following a gym workout with a trainer.

### Relevance for Clinical Practice

It is not only the behaviours of UHR individuals that should be targeted to promote a healthier lifestyle. Clinical services also need to be aware that monitoring physical health is important, and interventions to improve physical health in the UHR cohort are required. This arises from recent findings, which suggest physical health and associated health behaviours are not monitored on a routine basis by UHR services (Carney *et al.* 2015). Happell *et al*. (2012) also argue that despite being a rising topic, more research is required in order to facilitate integrated care for physical and mental health needs. One way to address this is to equip mental health nurses with the ability to promote physical health in this group.

The important role mental health nurses can have to improve the general health of people with mental health difficulties has previously been recognized (Bradshaw and Pedley, 2012; Happell *et al.* 2011; Robson and Gray, 2007) First, as many have daily contact with service users, this time could be used to work together to address unhealthy lifestyle factors (Bradshaw and Pedley, 2012; Stanton *et al.* 2015). Schemes, such as ‘Making every contact count’, might be effective, where health professionals are trained to facilitate behaviour change at every contact with a client (Lawrence *et al.* 2016). Second, nurses make up the largest component of the health‐care workforce, which results in an increased chance of implementation if interventions are rolled out on a larger scale. Third, encouraging mental health nurses to focus on both the physical and mental health of an individual promotes a more holistic approach to health care; one that is required to address physical health disparities (De Hert *et al.* 2011).

The outer circle of the behaviour‐change wheel focuses on changes to policy to encourage wider‐scale behaviour change, such as service provision, or policy guidelines (Table 1). Mandatory training in physical health promotion for all health professionals could allow it to be incorporated into general nursing practice. This could include training for mental health nurses to conduct physical health assessments and provide advice about diet, or integrating a physical health specialist into mental health services (Happell *et al.* 2016). Changes to policy guidelines might also be an effective way to promote physical health, such as including reminders to conduct physical health checks on the files of service users.

### Future Recommendations

Interventions to promote physical health are required for UHR individuals due to high rates of unhealthy lifestyle factors such as physical inactivity and substance use. We recommend mental health nurses and wider health professionals use the principles of the COM‐B model and behaviour‐change wheel to develop a new healthy lifestyle intervention for the UHR group. Developing an intervention using this systematic method may have advantages over traditional approaches used to promote healthy living in mental healthcare settings, which are often not linked to any underlying theoretical framework, or developed structure and are applied on an ad‐hoc basis. Due to limited existing research, further qualitative research should be conducted with this population and the clinical staff prior to developing any intervention to identify what behaviour would be the most appropriate target. Determining the barriers to and facilitators of healthy lifestyle in the group, and establishing which behaviours they wish to change (e.g. smoking, diet, and physical activity) will be useful. The perspective of mental health professionals should also be considered to determine which interventions they believe are feasible and acceptable approaches to use.

## Conclusion

Training mental health nurses and other health professionals to use the COM‐B model and behaviour change wheel could promote physical health for young people at risk for psychosis. Using a systematic and theoretically‐based approach to intervention development could result in effective methods of health promotion in this group. Given the lack of physical health research with the UHR group, we suggest that using a theoretically‐based framework to develop an intervention will provide a good baseline to conduct further research and develop health service provision for this population. The COM‐B model could be an appropriate framework to use, given the flexibility of the approach and ability to account for a wide range of behaviours. Additional training and development for mental health nurses could encourage a greater integration of mental and physical health care for young people in clinical services.
